# Hybrid Deep Learning and Discrete Wavelet Transform-Based ECG Biometric Recognition for Arrhythmic Patients and Healthy Controls

**DOI:** 10.3390/s23104635

**Published:** 2023-05-10

**Authors:** Muhammad Sheharyar Asif, Muhammad Shahzad Faisal, Muhammad Najam Dar, Monia Hamdi, Hela Elmannai, Atif Rizwan, Muhammad Abbas

**Affiliations:** 1 Department of Computer Science, COMSATS University Islamabad, Attock City 43600, Pakistan; sp21-rcs-010@cuiatk.edu.pk (M.S.A.); shahzad_faisal@ciit-attock.edu.pk (M.S.F.); 2 Department of Electrical and Computer Engineering, Air University, Islamabad 44000, Pakistan; 3 Department of Information Technology, College of Computer and Information Sciences, Princess Nourah bint Abdulrahman University, P.O. Box 84428, Riyadh 11671, Saudi Arabia; MSHamdi@pnu.edu.sa (M.H.); hselmannai@pnu.edu.sa (H.E.); 4 Department of Computer Engineering, Jeju National University, Jejusi 63243, Republic of Korea; 5 Department of Computer and Software Engineering, National University of Sciences and Technology, Islamabad 44000, Pakistan; m.abbas@ceme.nust.edu.pk

**Keywords:** ECG biometric recognition, deep learning, transfer learning, 1D convolutional recurrent neural network, DWT features

## Abstract

The intrinsic and liveness detection behavior of electrocardiogram (ECG) signals has made it an emerging biometric modality for the researcher with several applications including forensic, surveillance and security. The main challenge is the low recognition performance with datasets of large populations, including healthy and heart-disease patients, with a short interval of an ECG signal. This research proposes a novel method with the feature-level fusion of the discrete wavelet transform and a one-dimensional convolutional recurrent neural network (1D-CRNN). ECG signals were preprocessed by removing high-frequency powerline interference, followed by a low-pass filter with a cutoff frequency of 1.5 Hz for physiological noises and by baseline drift removal. The preprocessed signal is segmented with PQRST peaks, while the segmented signals are passed through Coiflets’ 5 Discrete Wavelet Transform for conventional feature extraction. The 1D-CRNN with two long short-term memory (LSTM) layers followed by three 1D convolutional layers was applied for deep learning-based feature extraction. These combinations of features result in biometric recognition accuracies of 80.64%, 98.81% and 99.62% for the ECG-ID, MIT-BIH and NSR-DB datasets, respectively. At the same time, 98.24% is achieved when combining all of these datasets. This research also compares conventional feature extraction, deep learning-based feature extraction and a combination of these for performance enhancement, compared to transfer learning approaches such as VGG-19, ResNet-152 and Inception-v3 with a small segment of ECG data.

## 1. Introduction

Human recognition is essential for many civil, military and scientific applications such as airport security, monitoring and criminal investigation, among others. Outdated attempts for recognizing individuals in previous years heavily relied on the items one carried, such as keys, passports, RFID cards, etc., or on what one knew, such as PINs or passwords, etc. Keys and cards, on the other hand, can be lost or stolen, whereas PINs and passwords could be forgotten or hacked. Biometrics were employed to close such security gaps and weaknesses [1].

Biometric data are a person’s unique property that would be dependent on what the individual owns and cannot be lost, forgotten, or stolen. Thus, there is no reason to worry about identification privacy or remembering complex passwords. Biometric technology can therefore be used to address the increasing demand for global security. Measurements refer to measures, while bio relates to life. These constitute an automatic method for identifying an individual utilizing particular biometrics. They could be classified into two categories based on such traits: physiological character traits and behavioral character traits [2]. A person’s gait or walking style, keystrokes, signatures, as well as speech patterns, among several others, are based on behavioral biometrics. These traits depend on the way a person behaves. Similarly, physiological traits belong to the physical structure and are intrinsic to the human body. This category includes body parts such as the ear shape, hand geometry, iris, face and fingerprint. Medical biometric technology is a part of physiological traits that include physical properties associated with the medical field and are particularly useful in patient identification and data integrity in the medical industry. Heartbeats, DNA, and other biological features are included. Electrophysiological signals such as ECG and EEG are examples of medical biometrics. Electrocardiograms can provide a more reliable biometric solution, even though they fulfill the majority of the requirements for an ideal biometric, especially when compared to other biometrics [1]. Because of the distinct variations in the morphology or structure of hearts, each person’s ECG signal has a unique pattern. Even twin brothers who have the same iris structure or facial features would have different ECG readings [1]. ECG offers the advantage of an innate property of liveness detection, which sets it apart from other biometric techniques. Liveness detection ensures that the individual providing the biometric is genuinely present and carrying it. It can significantly reduce the likelihood of fraud or other spoofing attacks on the biometric system [1]. Identifying individuals using medical biometrics is a critical task. In arrhythmia patients, for example, the flow of the heartbeat is irregular when using ECG signals. The impact of arrhythmia on an individual will be discussed in later sections.

The rate or rhythm of the heartbeat provides the defining feature of the heart disorder known as arrhythmia. A variety of heartbeat patterns exist, including those that are irregular, too fast, or too slow. Unlike bradycardia, which is associated with extremely slow heartbeats, tachycardia is a condition where the heartbeat is too fast [3].

To deal with patients with arrhythmia and other databases with irregular heartbeats, such as ECD-ID [4] and MIT-BIH [5], deep learning infrastructure techniques with adaptive feature extraction and intrinsic classification systems have recently gained popularity. However, these techniques usually require a large amount of training data. The paper [6] describes a technique for transfer learning that employs convolutional neural networks. This technique involves integrating information from the GoogLeNet [7] model, which was used for identification, pretraining the model with the “fine-tune” concept, and adding three adaptive layers behind the initial feature layer. An ensemble method of pretrained models was then trained for ECG signal classification using the ECG signal. The backbone consisted of two different VGG-16 [8] models and an Inception-ResNetV2 model with feature extracting layers and ImageNet weights [9]. The preprocessing of input ECG signals was accomplished using image processing techniques. The deep transfer learning method was used for feature extraction, and SVM was used for the identification of subjects [10].

The difference between deep learning and conventional methods is that deep learning techniques automatically learn intrinsic patterns from big data, whereas conventional methods heavily rely on designers’ prior knowledge and make it practically impossible to take advantage of large amounts of data [11]. However, in the case of conventional feature extraction techniques such as the discrete wavelet transformation (DWT), they retrieve valuable information in the time–frequency domain and represent a promising method for extracting the features from heartbeat responses. DWT generates a significant number of coefficients to describe the signal details and approximations at different scales. Therefore, selecting coefficients requires minimizing the feature size [12]. Consequently, deep network techniques extract useful data from raw information, and the extracted data are both relatively small and better than the original information, whereas conventional features such as DWT analyze data in the time and frequency domain to extract intrinsic features by decomposing the data on multiple scales.

The major contributions of this research are presented below

1. Integration of discrete wavelet transforms with one-dimensional convolutional recurrent neural networks at the feature level to improve the ECG biometric recognition rate.
2. This research compared the proposed methodology with the transfer learning of pretrained architectures such as [8], ResNet-152 [13] and Inception-V3’s [14].
3. Performed analysis on a combination of all publicly available datasets of NSRDB [4], ECGID [4] and MIT-BIH [5] with a total of 156 subjects, including arrhythmic patients and healthy controls.

This research introduces Deep-Hybrid-ECG, a combination of deep learning and discrete wavelet transform-based approaches for ECG biometric recognition. The biometric identification was performed with a feature-level fusion of one-dimensional convolutional recurrent neural networks (1D-CRNN) and discrete wavelet transform (DWT). The approach has never been employed in previous research studies, to the best of our knowledge. The paper is structured as follows. Section 2 examines the review of the literature, the proposed methodology is presented in Section 3, and Section 4 discusses the experiment’s findings. Furthermore, finally, Section 5 wraps up the research with future implications.

## 2. Related Work

This section discusses the research so that our computational system can be compared to similar works. In the study by Wang et al. (2019), the time and frequency information of the raw data, learned from the DWT feature, was referred to as temporal-frequency autoencoding. This approach achieved high accuracies in identifying multiple heartbeats: 98.87% on raw electrocardiogram (ECG) data from ECG-ID (two-recording) [4], 92.30% on ECG-ID (All-recording) and 96.82% on MIT-BIH-AHA [5] databases. This study [15] is based on information extracted from four distinct 15-minute-long RFII recordings, each related to a different subject. The above system achieved recognition rates of up to 99.9% using the CWT approach and rate increases of up to 100% using the DWT approach. The ECG data was collected by repeatedly recording 30 healthy individuals performing six regular everyday activities at a sample rate of 1000 Hz. The QRS segment was extracted using discrete wavelet transform (DWT) algorithms, including the first three Daubechies (dB) discrete wavelets and the Cardioid graph. To validate the matching process, classification was performed using the multilayer perceptron (MLP) technique. In [16], even when the data file was compressed to 73.33%, the recognition rate reached 96.46%. The study by Belgacem et al. [17] was used to identify 80 individuals from four publicly available ECG databases (MIT-BIH [5], ST-T, NSR [4], PTB) and an electrocardiogram dataset collected from 20 students at Paris Est Institution. For feature extraction, the discrete wavelet transform (DWT) was used. The random forest framework presented for the ECG signal authentication was accurate and achieved a low error rate and 100% subject detection accuracy for healthy individuals using a reduced number of features. In this attempt, the feature vector from [1] was extracted by first extracting the heartbeat and then employing the discrete wavelet transform (DWT) to retrieve the wavelet coefficients. The method was evaluated on public datasets such as the MIT-BIH/arrhythmia (MIT-BIH [5]), MIT-BIH-Normal Sinus Rhythm (NSRDB) [4] and the ECG-ID database (ECG-IDDB) [4], with achieved accuracies of 93.1%, 99.4%, 82.3% and 94.4%, respectively. For this study [18], BIOPAC mechanisms were used to collect ECG signal data. The SS2L electrode Lead Set and body surface electrodes were used to collect ECG data. The dataset includes ECG recordings from fourteen subjects at rest, eight men and six women. Each ECG recording was sampled at 1 kHz. To remove artifacts or extract regions of focus from raw ECG signals, empirical mode decomposition (EMD) was used, and the features were extracted by obtaining indicative attributes from the frequency, time and statistical domains. SVM with a cubic kernel achieved the highest accuracy of 98.7%, sensitivity of 100% and specificity of 98.8% for the successful identification of fourteen subjects.

The system is sensitive to the local distinction among heartbeats from different subjects, according to the author [19], because it utilizes principal component analysis filters as the convolution kernel. Finally, linear (SVM) is applied to address the identification for faster classification and training [20]. With only five heartbeats from 12 subjects, the proposed strategy achieves greater subject identification accuracy (94.4%) on ECG signals using the ECG-ID [4] Database. The author of [21] employs a convolutional neural network to acquire complicated patterns in data directly from electrocardiogram image representations, eliminating the requirement for extensive extracting features. The detection rate from two datasets was 98.4% (one-arm ECG) or 91.1% (two-arm ECG) (ear ECG). A deep learning-based 1D-CNN was used in this [22] attempt to categorize ECG data for biometrics. The NSRDB and ECG-ID [4] databases are used in the experiments. The accuracies of the NSRDB and ECG-ID databases are 96.93% and 100%, respectively. Machine learning and deep-learning algorithms were used to extract features from ECG signal intervals. SVM as well as other machine learning methodologies’ deep learning are built on CNN and employ ANN. Because of their different ECG behaviors, MIT-BIH [5], FANTASIA, NSRDB [4] and QT between the datasets can be considered. The suggested convolutional neural network approach in [23] obtained accuracies of 81.33%, 96.95%, 94.73% and 92.85% for the MIT-BIH [5], FANTASIA, NSRDB [4] and QT databases, respectively. In feature selection, Ref. [24] proposed a deep neural network (DNN) entirely based on a denoising auto-encoder (DAE). Recognition rates of 98.10% and 95.67%, respectively, were obtained on self-collected and high-pressure data, and 94.39% on a merged MIT-BIH database and self-collected data. The [23] study’s goal was to combine convolutional neural networks with a supervised back-propagation algorithm learning technology to identify an individual based on fiducially extracted features from an ECG Signals LEAD-I signal. The accuracies achieved utilizing 3, 10 and 20 classes on the ECG-ID [4] dataset were of 98.24%, 96.24% and 94%, respectively. The author proposed a new identification method in [25] based on electrocardiograms (EKGs) transformed into a heatmap of a collection of aligned R-peaks (heartbeats), which tends to result in an electrocardiomatrix. A simple CNN model with high accuracy and a low bit error rate is used as a verification solution to illustrate the feasibility of using EKMs (obtained from ECG traces) for identification. The NSRDB was 99.53% accurate, with a false acceptance rate of 0.02% as well as a false rejection rate of 0.05%. The MIT-BIH and PTB datasets produced very similar results. The system described in [26] employs advanced deep learning methods worn on a single arm to learn from high-level features in ECG raw data. The ECG signal was converted into the two-dimensional domain for CNN to learn the ECG Signals data. Using single-arm ECG, an accuracy of 98.4% was achieved in this study.

The concept of transfer learning was used in [27] to fine-tune a PlexNet model using pretrained ResNet and DenseNet models. The proposed ensemble for human identification was tested on publicly available datasets such as PTB and CYBHI, demonstrating its effectiveness with an identification accuracy of 99.66% achieved on both healthy and unhealthy subjects. In [9], two modified VGG-16 approaches and one InceptionResNetV2 design with an additional feature extraction layer and ImageNet weights were proposed. After 5-fold cross-validation with the MIT-BIH normal sinus rhythm database and BIDMC datasets, the model achieved 99.98% accuracy. Ref. [10] suggests an ECG feature framework for human authentication with a cancelable ECG method. A transfer learning method was used to extract ECG features, followed by a recommended cancelable technique to defend deep network templates. Finally, a support vector machine (SVM) achieved 99.26% accuracy on the ECG-ID dataset and 99.39% on the PTB dataset for authentication. In [28], several machine learning methods such as the SVM of different kernel parameters and deep neural network designs were used to identify the ECG-ID dataset of QTc augmentation (e.g., RNN and CNN). As a result, the classifier performance was compared with and without QTc augmentation.

The study [29] used the ASCERTAIN database, which included five personality scale items and emotional self-assessments from 58 users, along with data from electro-encephalographies, electrocardiograms, galvanic skin responses and facial activity. The scheme for transforming electrocardiogram signals into various wavelets and then applying them to the ensemble deep learning model outperforms CNN or ResNet18 techniques. The study had a 98.10% success rate. This research proposed an ECG identity verification technology based on the method in [6], which relies on the recurrence graph and transfer learning. The recurrence plot was used to create two-dimensional images from one-dimensional time series. The GoogLeNet model was modified for ECG identification, achieving 96.26% accuracy on the PhysioNet D1 database. DenseNet, Xception and ResNet are well-known convolutional neural network models that were used in this study by [30]. For evaluation, the PTB-ECG dataset was used, and the accuracy obtained by the opened CNN model with a spectrogram was 98.85%, ResNet101 was 97.95%, and DenseNet201 was 98.99% with MFCC. Numerous available public ECG and biometric databases were used to test the system. The goal of the research in [31] was to develop a secure multi-modal biometric technology using a different fusion level of convolution neural network (CNN) as well as the Q-Gaussian multi-support vector machine (QG-MSVM). That method was 99.83% accurate. In this study, the proposed technique in [32] was tested on the PTB-ECG and CU-ECG datasets. PTB identification rates for ResNet, DenseNet, VGGNet and Xception were 97.02%, 97.33%, 97.83% and 97.16%, respectively, and the CU-ECG identity rates were 90.56%, 93.49%, 92.11% and 92.8%, respectively.

The model [33] was evaluated against two publicly available datasets, the ECG-ID data (ECGID) [4] and the MIT-BIH arrhythmia database [5]. According to the experimental results in [33], the proposed method using the BGRU design, which is a bifacial mixture of the recurrent neural network and GRU cell units, achieved a high classification accuracy of 98.55%.

The system was built using deep learning algorithms such as convolutional neural networks (CNNs) and a long short-term memory (LSTM) architecture with a customizable activation function. The authors in [34] evaluated the proposed method with off- and on-person datasets such as ECG-ID, PTB, CYBHi and UofTDB, and obtained accuracies of 99.49%, 99.62%, 94.86% and 95.36%, respectively. A methodology that relies on LSTM was created in this research [35] for human recognition using an ECG signal. The MIT-BIH arrhythmia [5], CYBHI, ECG-ID [4] and PTB databases have all been thoroughly tested to ensure the model’s efficacy. The accuracy for 290 persons in the PTB database is 97.3%. Other databases produce similar results, with CYBHi obtaining an accuracy of 79.37%. Using a BLSTM network, the suggested data-independent approach in [36] achieves a relatively better identification accuracy for each database. The proposed technique in [37] was tested using deep learning methods such as CNN and LSTM on self-collected databases S1 and S2, with the experimental results showing an EER% of 0–10.13% for the S2 database and the mixed S2 and S1 datasets, 0–5.31% for one-day enrollment, and 0.033–3.93% and 0–1.35% for two-day enrollment. The study in [38] proposed using an electrocardiogram and an ensemble of CNN and LSTM for personal identification, using the CU-ECG database created by Chosun University, which showed an accuracy greater than 90% in identifying individuals based on ECG signal characteristics. The proposed method in [39] used a 1D CNN model to eliminate incorrect heartbeats from low-quality ECG recordings, while an attention-based bidirectional LSTM was employed to learn high-level identification features. Experimental results on the publicly available ECG-ID [4], AHA [5], LT-AF, AFDB, STAFF-III and MIT-BIH datasets showed accuracies of 98.84%, 99.2%, 97.07%, 99.5%, 97.94% and 97.39%, respectively, with 97.54% and 99.70% accuracy obtained on ECGID and the MIT-BIH arrhythmia datasets, respectively.

In [40], the data are transformed from the temporal domain into the wavelet domain, and then a parallel 1D-CNN is employed to automatically discover multiscale feature hierarchical structures from the raw wavelet data. The model performed well on eight ECG datasets, with an accuracy rate of 93.5%. In [41], an EMG-based individual identification strategy based on continuous wavelet transform and Siamese systems was proposed. The MYO armband from Thalmic Labs was used to capture EMG signals. Experiments show that, using this method, the identification results for 21 subjects can reach 99.285%. In [42], techniques are proposed for recognizing humans using ECG based on a combination of discrete wavelet transform, continuous wavelet transform and a novel deep learning approach called the capsule network.

The accuracy on the MIT-BIH arrhythmia [5], PTB STDB and NSRDB [4], respectively, was 99.5%, 98.1%, 98.2% and 100%. Methods for feature extraction including such a Dwt wavelet (‘db8’, ‘db3’ and ‘db10’), Biorthogonal wavelet ‘bior2.6’ and symlets wavelet ‘sym7’ are used in [43] this work. A backpropagation (BP) neural network and radial basis function neural network are combined to serve as a classifier. The proposed method obtained an identification rate of 98.41% when using the Daubechies wavelet ‘db8.’ The proposed techniques were evaluated on some other 250 heartbeats after being trained on 250 heartbeats chosen at random. In those situations, the average identification accuracy for CWT and STFT is 96% and 94.72%, respectively, as achieved in [44]. Before being served into the transfer learning approaches, the Electrocardiogram signal was transformed into scalogram image data that use the continuous wavelet transform. This paper [45] evaluates AlexNet, GoogLeNet and ResNet. ResNet was 0.73–0.27% higher on PTB-ECG than AlexNet or GoogLeNet and 0.94–0.12% higher on CU-ECG than GoogLeNet or AlexNet. A pretrained network light CNN (GoogLeNet) was used to test and train the continuous wavelet transform images to extract from the segmentation process. On the PTB dataset, the method in [46] achieved 99.83% accuracy on light CNN and 99.94% accuracy on GoogLeNet.

To enhance the performance of the electrocardiogram (ECG) biometric identification method, we introduce feature-level fusion using a one-dimensional convolutional recurrent neural network and discrete wavelet transform in our proposed method. Our model’s performance was evaluated using combined datasets such as ECG-ID, MIT-BIH and NSRBD, all of which are publicly available on the PhysioNet website. In this study, pretrained transfer learning methods such as VGG-19, ResNet-152 and Inception version 3 are used to compare the results of a one-dimensional recurrent convolutional neural network (1D-CRNN) and discrete wavelet transform-based concatenated features. Section 3 and Section 4 cover the methodology and results of the experiment.

## 3. Material and Methods

The databases are discussed in detail in the section below:

### 3.1. Materials

#### 3.1.1. ECG-ID Database

There are 310 electrocardiogram (ECG) recordings from 90 individuals in the database. Each recording includes 20 s of electrocardiogram (ECG) lead I with 12 bits digitized at 500 Hz resolution over a nominal 10 mv range. Volunteers supplied the data for the records (46 women and 44 men aged between 13 of 75 years old which comprised the authors’ colleagues, students and friends). Each person’s record collection ranges from 2 (collected in 1 day) to 20 (collected periodically in 6 months). The raw electrocardiogram (ECG) signals are noisy, with components of high- and low-frequency noise.

#### 3.1.2. MIT-BIH Arrhythmia Database

A list of patients who suffer from arrhythmia can be found in the MIT-BIH Arrhythmia Database. An irregular pulse characterizes a heart arrhythmia. Heart arrhythmias (problems with the heartbeat) happen whenever the electrical signals which support the heartbeat may not properly function. The faulty flag leads the heart to collapse prematurely, moderately, or unexpectedly. The MIT-BIH Arrhythmia Database includes 48 half-hour ECG recordings from 48 subjects studied by the BIH Arrhythmia Research Institute between 1975 and 1979. At Boston’s Beth Israel Clinic, electrocardiogram (ECG) recordings were collected from a combined population of outpatients (approximately 40%) and inpatients (approximately 60%). The recordings were tested per channel per second and digitized at 360 Hz, including an 11-bit resolution in a 10 mv range.

#### 3.1.3. MIT-BIH Normal Sinus Rhythm Database

This dataset includes 18 long-term electrocardiogram (ECG) recordings of persons referred to the Arrhythmia Research Facility at Boston’s Beth Israel Clinic. There are no critical arrhythmias found in the participants in that dataset which included 13 women aged 20–50 and 5 men aged 26–45. The recordings had digitized at 128 Hz per channel including an 11-bit resolution at a 10 mv range. The details of databases are shown in Table 1.

### 3.2. Proposed Methodology

**Figure 1:**
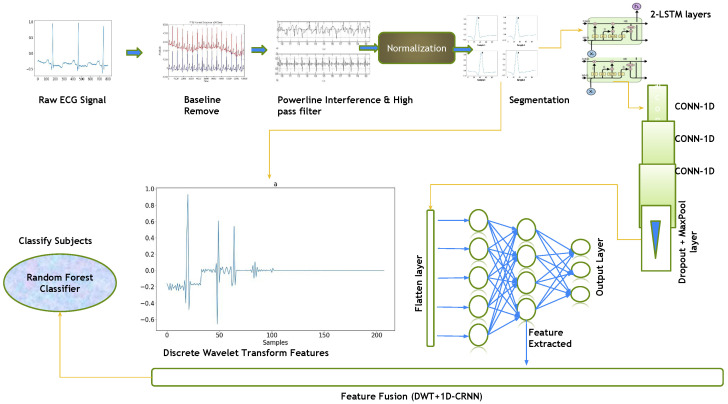
Flow diagram of the complete methodology, including preprocessing and segmentation steps, feature level fusion of DWT and 1D-CRNN.

This paper proposes a hybrid features-based model that includes the discrete wavelets transform (DWT) and the one-dimensional convolutional recurrent neural network (1D-CRNN). These hybrid features were used to classify people by ECG signals. The proposed architecture was divided into four stages: preprocessing ECG signals, feature extraction using DWT and 1D-CRNN, feature concatenation and classification. The raw ECG signal is normalized and segmented on the bases of R-peaks, and then, 1D-CRNN and DWT are applied to the segmented signal for feature extraction, followed by the concatenation of both DWT and 1D-CRNN features. As shown in Figure 1, the concatenated features are forwarded to the Random Forest Classifier (RFC), which represents the features to their class labels based on the expected likelihood resemblance.

### 3.3. ECG Signal Preprocessing

ECG preprocessing involves steps including baseline removal, noise removal and heartbeat segmentation.

#### 3.3.1. Baseline Removal

The baseline wanders the voltage at which all cardiac muscle cells are at rest. The subject’s breathing, movement, or electrode displacement causes baseline wander, which is a low-frequency noise. As a result, amplitude shifts make the detection of the R peak more difficult. The baseline signals are removed from the following databases, the average value of the signal at the first stage is calculated, and the average value from the signal is subtracted. The equation of baseline removal is given below [47]:

(1) $$mean_{ECG}=\frac{1}{N}\left(\sum_{i=0}^{n}ECG\right)$$

(2) $$new_{sig}=ECG-mean_{ECG}$$

where ECG denotes the original signal and *N* indicates the total number of data points in the signal. The $mean_{ECG}$ value was calculated by dividing the sum of all data points by the total number of data points. The original signal is then subtracted from the value of the $mean_{ECG}$ variable, which is then saved in the $new_{sig}$ variable.

#### 3.3.2. Powerline Interference

A band reject filter is used to remove or attenuate the powerline interference present in the ECG signal. The band frequency is typically 50 Hz or 60 Hz, depending on the country. Therefore, the equation for a band reject filter for ECG signals can be written as:

(3) $$P\left(f\right)=\frac{1}{\left(1-2r\cos\left(\frac{2\pi f}{fs}\right)+r^{2}\right)}$$

where:

- *r* is the filter pole radius, which determines the notch frequency;
- *f* is the frequency in Hertz;
- *fs* is the sampling frequency in Hertz;
- cos() is the cosine function.

Using the Neurokit [48] tool, the powerline interference with a frequency cutoff value of 50 Hz and an order of 5 is removed.

#### 3.3.3. High Pass Filter

An ECG signal has a frequency range of approximately 0.5 Hz–85 Hz. Alternating current fields in the patient’s cable caused distortion. Using a Butterworth technique A high-pass filter was applied to remove the noise with a frequency cutoff value of 1.5 Hz. The frequency response of this filter is shown below:

(4) $$H\left(f\right)=\frac{f}{\sqrt{\left(1+f^{2/2.5\times 10^{-4}}\right)}}$$

where *f* is the frequency in Hz. Note that the gain of the filter increases with increasing frequency, reaching a value of 0.707 (−3 dB) at the cut-off frequency of 0.5 Hz. This means that frequencies below 0.5 Hz are attenuated by the filter, while frequencies above 0.5 Hz are passed through. Using a high-pass filter with a cut-off frequency of 0.5 Hz, the low-frequency noise and baseline wander can be removed from the ECG signal. Using the Neurokit [48] tool, low-frequency noise is removed.

#### 3.3.4. Normalization Using Min-Max Scaler

**Figure 2:**
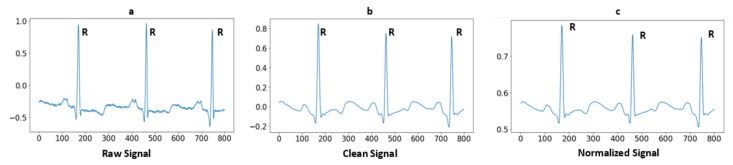
Signal preprocessing steps: (**a**) Raw ECG signal; (**b**) After applying high-pass filter and powerline interference; and (**c**) After normalization of signal.

In the last step of the preprocessing, the signal is scaled from the range of −1–1. The equation for the min–max scaler is

(5) $$N_{i}=\frac{S_{i}-\min\left(S_{i}\right)}{\max\left(S_{i}\right)-\min\left(S_{i}\right)}$$

where $N_{i}$ represents the normalized signal and $S_{i}$ represents a clean signal. According to the Equation, Ref. [1] divided the difference between both the minimum and maximum $S_{i}$ values And subtracted a minimum $S_{i}$ value from a cleaned signal $S_{i}$. The array values are normalized from −1 to 1. Figure 2 illustrates the raw signal without cleaning after applying baseline removal, a powerline interface and a high-pass filter, so the signal is clean-formed. Furthermore, the last one is a normalized signal for use as a feature.

### 3.4. Segmentation

**Figure 3:**
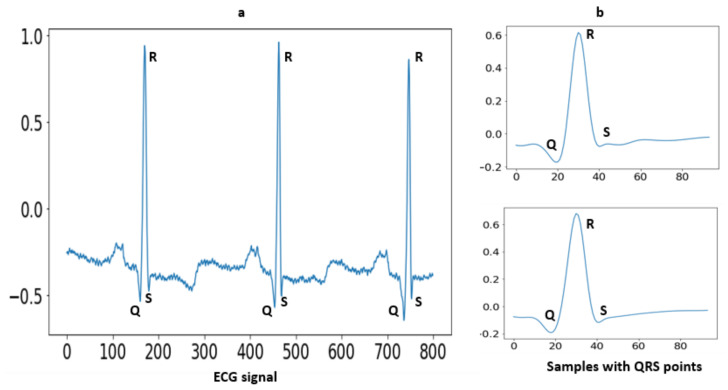
(**a**) Raw ECG signal with fiducial points; (**b**) The extracted QRS complex with 45 samples before R-peak and 49 samples after R-peak.

**Figure 4:**
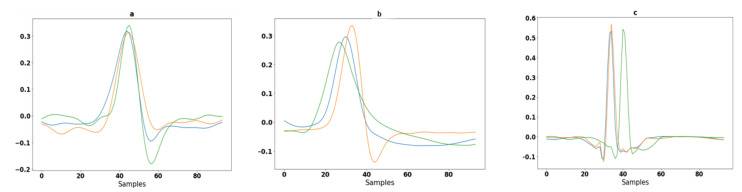
Segmentation of ECG signals in different colors shown for three randomly selected subjects: (**a**) 94 samples extracted from ECG-ID database from random subjects; (**b**) 94 samples extracted from MIT-BIH database from random subjects; and (**c**) 94 samples extracted from the NSRDB database from random subjects.

Segmentation is an approach in which some portion of data is extracted from the signal that is used for further evaluation purposes. In the case of MIT-BIH arrhythmia, some ECG signals higher T-peaks than R-peaks. After removing baseline the drift as well as low- and high-frequency noise, the signals are in a form to detect the R peaks. A well-known Pan–Tompkins [49] method is used for R Peaks detection. In the literature [19], Pan–Tompkins is already applied for R-Peaks detection for the ECG-ID, MIT-BIH and NSRDB databases. The electrocardiogram (ECG) is an interpretation of the QRS complex. The R point is the local maximum point shown in Figure 3, the Q point is on their left side and the S point is on their right side. One of the most important sections of this complex is the R wave. A total of 94 samples are extracted from the signal using the R-peaks point. These samples are fed to a convolutional recurrent neural network (1D-CRNN) and discrete wavelet transform (DWT) for analysis. The QRS complex samples shown in Figure 4 and the equation for segmentation are given below [1];

(6) $$S_{r}=\left[S\left(R_{p}-45\right):S\left(R_{p}-49\right)\right]$$

where $S_{r}$ saves the segmentation results from 49 samples after the peak and 45 samples before the R-peak and $S(R_{p})$ represent the peak points in the signal. Given the fact that Equation (4) is repeated for each signal sample,

### 3.5. Proposed CRNN Architecture

ECG comprises time series data and LSTM is particularly useful for time series and sequential data. The proposed CRNN model can also exploit consecutive QRS with repetitive patterns. Even for a single QRS segment, the use of LSTM is significant for sequential ECG data, comparing it with conventional signal processing-based feature extraction techniques that struggle with complex temporal dependencies. ECG-segmented samples are passed as input to the CNN architecture. Each sample has 94 values, the sample has been reshaped to (1 × 94) for passing to the convolutional neural network. This is a one-dimensional data single-column with 94 values, so the input shape for CRNN is (1 × 94). The CNN architecture made with the combined LSTM and 1D convolutional layers. The one-dimensional convolutional neural network accepts dimensional data with the shape of 1 × 94. There are two LSTM layers the input has passed to the LSTM with hidden unit 128 and the second LSTM layer with hidden unit 64 and the return sequence should be true to obtain a 1D array to pass the next layer. The two consecutive LSTM layers can improve the model’s ability to capture complex temporal dependencies in the ECG signal and mitigate the vanishing gradient problem. The low-level features and patterns in the time series data of ECG are learned with the first LSTM layer, and capture higher-level features and dependencies using a second layer. The use of stacked LSTM layers for time series data can potentially increase the performance of the classification task [50,51].

**Figure 5:**
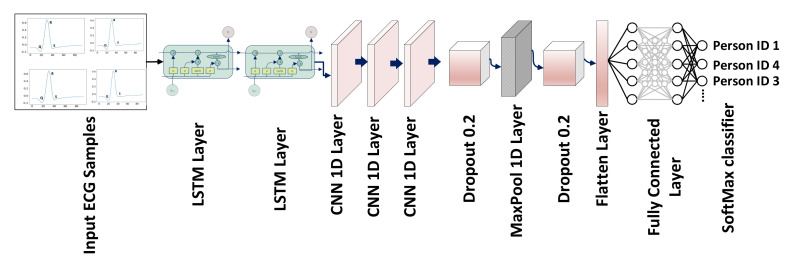
CRNN network with LSTM, 1D convolutional, dropout, flattened and FC layers, as well as the applied activation function, which is SoftMax for classification.

There are three 1D convolutional layers consecutively, the first convolutional layer consists of 32 filters of kernel size 3, the second convolutional layer consists of 64 filters of kernels of size 5 and the third convolutional layer consists of 32 filters of kernels of size 8 with the same padding values and the activation function of ReLU. A dropout layer of 0.5 followed by convolutional layers discards 20% of random features to avoid overfitting. A MaxPool layer has applied after the dropout layer of size is 5 with a stride of 2 and then a flattened layer is added to convert features into a single array. Three layers were fully connected followed by the flattening layer, the FC1 and FC2 units of 256 and 128 with the activation function ReLU and the FC3 unit according to the number of classes with the activation function SoftMax applied for classification. Figure 5 depicts the network’s architecture. The first two LSTM layers have learnable parameters of 66,560 and 49,408; the first, second and third convolutional layers have learnable parameters of 6176, 10,304 and 16,416; and the final 2 fully connected dense layers have learnable parameters of 32,896 and 6192. Figure 5 and Table 2 includes a thorough discussion of the 1D-CRNN architecture. The Dense 1 layers is used as a feature layer, and the extracted number of features is 128 because the layer’s output shape is 128. These 128 features were concatenated to DWT for classification. The proposed model is based on feature level fusion, not the decision level fusion. The last layer of 1D-CRNN is the output fully connected layer that can classify the person according to the label. For feature level fusion, we removed the fully connected layer, then extracted a feature from the second last layer that contains 128 values, which was used as a feature vector to concatenate with the DWT features. 

### 3.6. Discrete Wavelet Transform (DWT)

The DWT has been used to obtain discriminate hybrid features for classification. The discrete wavelet transform (DWT) can analyze complicated biomedical signals in both the time and frequency domains. The signal decomposition using the discrete wavelet transform (DWT) provides information about lower and higher frequency ranges for long and short time intervals. Because ECG signals seem to be short-term in nature and vary in frequency over time, FT and FFT do not examine the signal as well as those that can analyze the signals in the frequency domain. The fast Fourier transform (FFT) is made up of sines and cosines, whereas the electrocardiogram (ECG) contains information in both the time and frequency domains [1]. The discrete wavelet transform (DWT) has properties that allow it to analyze signals in both the time and frequency domains, with multiple resolution levels called multiresolution analysis. By increasing the value of decomposition or resolution, a better estimation of the signal was obtained concerning its length. The wavelet’s general form equation is given below.

(7) $$\psi \left(t\right)=\sum_{i=-\infty }^{\infty }h_{\psi }\left(n\right)\sqrt{2}\phi \left(2t-n\right)$$

**Figure 6:**
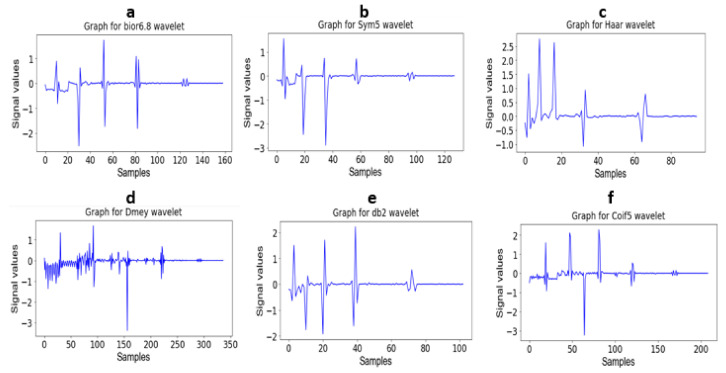
(**a**): Decomposition of signal using bior 6.8 wavelet (**b**): Decomposition of signal using Sym5 wavelet (**c**): Decomposition of signal using Haar wavelet (**d**): Decomposition of signal using Dmey wavelet (**e**): Decomposition of signal using db2 wavelet and (**f**): Decomposition of signal using Coif5 wavelet.

The wavelet coefficients were retrieved as a feature map for further processing. The mother wavelet with the scaling function was used for signal decomposition into multiple levels, which is known as wavelet decomposition, and the analysis of this method is called multiresolution wavelet analysis. Figure 6 depicts the different family wavelets obtained through multilevel decomposition analysis.

### 3.7. Features Extraction Using Transfer Learning Models

The three pretrained models are used for the evaluation of ECG biometric recognition to compare with the proposed methodology. The included models are VGG-19, ResNet-152 and Inception version 3.

#### 3.7.1. VGG-19

**Figure 7:**
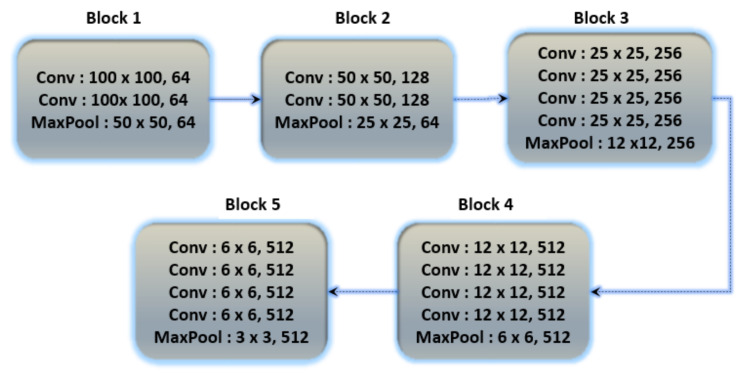
Basic architecture of VGG19 contains 5 blocks with multiple convolutional layers.

The architecture of the fine-tuned VGG19 model and the basic architecture of VGG19 is shown in Figure 7. The number of total learnable parameters is 59,604,784. The VGG19 consists of five convolutional blocks. In the first block, two convolutional layers with output shapes (100 × 100 × 64) with 1792 and 36928 trainable parameters, respectively, are connected to the MaxPooling layer with an output shape (50 × 50 × 64). In the second block, two convolutional layers are connected with the max-pooling layer, the output shape of the convolutional and max-pooling layers are (50 × 50 × 128) and (25 × 25 × 128) with 73,856 and 147,584 learnable parameters, respectively. The third block contains four convolutional layers with an output shape (25 × 25 × 256) and 295,168, 590,080, 590,080 and 590,080 learnable parameters, respectively, connected to the max-pooling layer of output shape (12 × 12 × 256). The fourth block consists of four convolutional layers with 1,180,160, 2,359,808, 2,359,808 and 2,359,808 learnable parameters, respectively, and the shape of the convolutional layers is (12 × 12 × 512) and afterwards, the connected max-pooling layer is (6 × 6 × 512). The fifth block contains four convolutional and max-pooling layers, the learnable parameters and output shape of all convolutional layers is 2,359,808 and (6 × 6 × 512) and the shape of the max-pooling layer is (3 × 3 × 512). Further layers are added to train the Dataset according to their output shape. The batch normalization layer normalizes the features of the above layer, and there are 3584 learnable parameters for the batch normalization layer. The global average pooling layer converts a three-dimensional into a one-dimensional array with an output feature length of 512. After the global average pooling layer, the four dense layers and one dropout layer are connected, the first two layers with 512 and 256 output units, respectively, and the last two layers with 256 and 128 output units and the dropout layer is applied between them. The accuracy score for that fine-tuned model is shown in Table 9.

#### 3.7.2. ResNet-152

**Figure 8:**
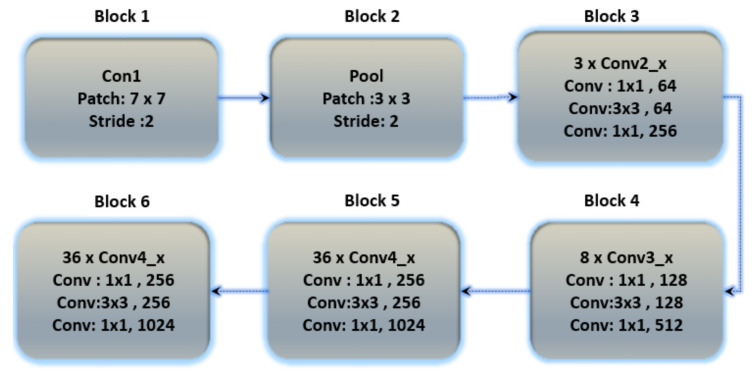
The basic architecture of the ResNet-152 model contains six blocks with different layers.

Residual networks (ResNets) have been proposed as a class of deep learning models with similar patterns but varying depths. To relieve the degradation of deep neural networks, ResNets introduced a structure known as the residual unit. The unit structure is just a feed-forward channel with a shortcut connection that adds new inputs as well as generates new outputs to the networks. The main advantage of this component is that it improves the accuracy of the model without increasing the model complexity. Figure 8 depicts the basic structure of ResNet-152, and additional layers were added to ResNet-152 for the classification of ECG databases in Table 9.

#### 3.7.3. Inception Version 3

**Figure 9:**
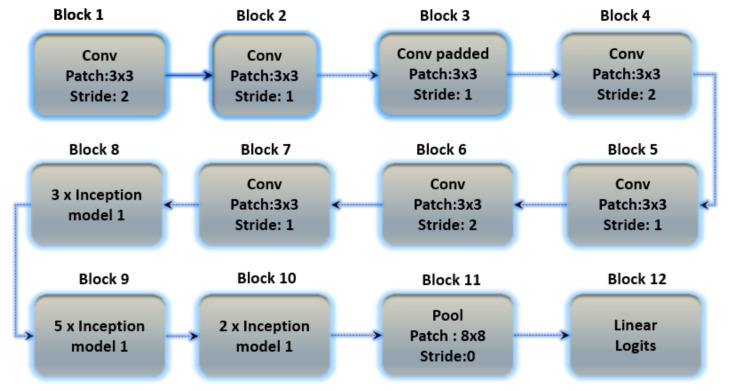
The basic architecture of the Inception V3 model containing 12 blocks with multiple layers.

Inception version 3 is the third version in Google’s deep learning convolutional architecture series. Inception version 3 was trained on a 1000-class dataset. Transfer learning allows users to train up the final layer of the existing design, resulting in significant time and database size savings. The Inception v3 model is a well-known transfer learning model. On some very powerful systems, the model trained millions of images from 1000 classes. Figure 9 depicts the architecture of Inception version 3.

## 4. Results

This section describes the results of the proposed research methods examined on the database in this section. The nature of both datasets is different for cross-section analysis, with two sessions out of each dataset being recorded. The dataset’s session contains the training and testing sets. The above sessions were trained on hybrid feature-based proposed architectures, including the proposed convolutional recurrent neural network (1D-CRNN), the discrete wavelet transform (DWT) model and the hybrid feature (DWT+1D-CRNN) model. The hybrid feature model means that the extracted features for DWT and 1D-CRNN are combined and passed to the classifier, as shown in Figure 1.

This research used a multi-class classification approach for the biometric identification. The following metrics were used to assess the functionality of the proposed architectures. False positive (FP), true positive (TP), false negative (FN) and true negative (TN) are examples (FN). The TN is an example of a situation in which an unknown input sample is not classified by the system, whereas when it has been classified into one of the classes, then FP occurs (Type I error). The TP is an example of a correctly classified sample from the dataset, whereas FN is an example of an incorrectly classified sample from the dataset (Type II error).

### 4.1. Conventional DWT Feature Extraction

The discrete wavelet transform (DWT) is constructed to demonstrate signals in both the time and frequency domains. The fundamental wavelet’s interpretation and extension shape it. The original signal has been approximated using the change coefficients. The analysis range in time is smaller when using a smaller scale; even so, within the frequency domain, it is equal to the use of the excessive frequency wavelets analysis method, i.e., using high-frequency wavelet decomposition for precise observation. A large scale allows for a wider analysis range in the time domain, but it is equivalent to employing a low-frequency wavelet for observations in the frequency domain. The DWT equation is presented in Section 3.3.4, Equation (5).

**Figure 10:**
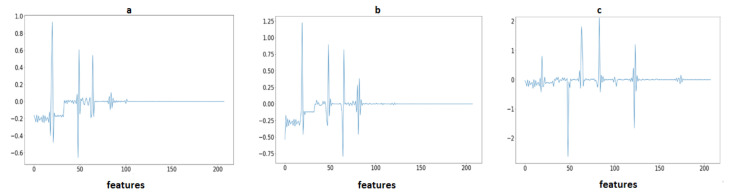
(**a**) The signal is decomposed into 4 levels and then all coefficients are concatenated for the ECG-ID database; (**b**) The signal is decomposed into 4 levels and then all coefficients are concatenated for the MIT-BIH database; (**c**) The signal is decomposed into 4 levels and then all coefficients are concatenated for the NSRDB database.

The maximum frequency of the signal, based on the sampling theorem, is $f_{s}/2$. When a signal is decomposed into order L using the Mallat algorithm, the corresponding frequency bands of the signal have decomposed into L + 1 subbands. The decomposition of the signal is shown in Figure 10.

For feature extraction, the signal is decomposed to the order of 4. The coefficients of the decomposed signal are used as a feature to pass the classifier. The result of conventional DWT features on the different DWT families and the classifier is shown in Table 3, Table 4 and Table 5.

After comparing the results of different classifiers for DWT families, it was found that the random forest classifier performed better than the others. This classifier has a hyperparameter called “n estimators” that determines the number of trees in the forest which was set to 100. Additionally, the random state option was used to ensure that the method produces the same results each time it is run, which is important for consistency. The random forest classifier is known for its high accuracy, ability to handle both categorical and continuous data. It also provides a ranking of features.

### 4.2. DWT and 1D-CRNN Feature Ranking

**Figure 11:**
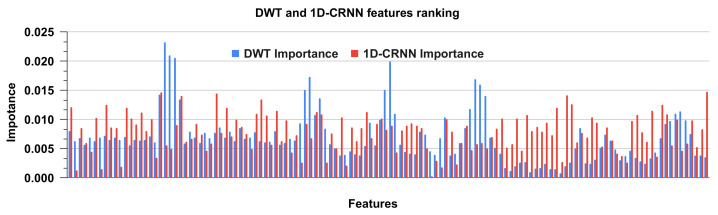
Ranking of DWT and 1D-CRNN features using the random forest classifier.

Random forest is a regression, classification and feature ranking ensemble learning approach. It is made up of decision trees that have been trained on random subsets of data and features. The relevance score is calculated to rank the importance of features by measuring the decrease in data impurity when a feature is used for tree splitting. Impurity can be measured using a variety of metrics such as Gini impurity or entropy. To obtain a more consistent assessment, the relevance score of each feature is averaged across all trees in the forest. This ranking can assist in identifying the most relevant features, which can then be used for feature selection or data reduction. The feature ranking for DWT and 1D-CRNN features is shown in Figure 11. The feature ranking is analyzed for DWT and 1D-CRNN on the combined dataset. The ranking of features shows the significance of both DWT and 1D-CRNN features, as well as the combination of these features.

### 4.3. CNN-Based Feature Extraction Using Transfer Learning

**Figure 12:**
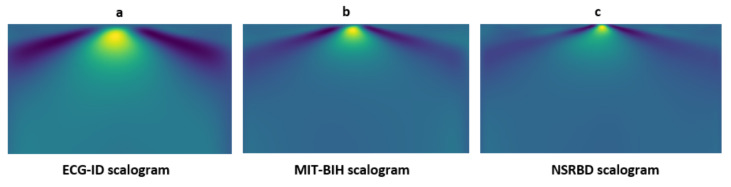
Signal preprocessing steps: (**a**) The ECG signal sample scalogram for the ECG-ID database; (**b**) The ECG signal sample scalogram for the MIT-BIH database; and (**c**) The ECG signal sample scalogram for NSRBD database.

The model was evaluated on the ECG signal to obtain results. In the initial stage, the signals are converted from a one-dimensional to a two-dimensional domain. For that purpose, the ECG signals are transformed into scalogram images with the help of continuous wavelet transform (CWT) and then passed to the pretrained models. The scalogram images are shown in Figure 12, and all the samples of the mentioned datasets are randomly split into 20% testing data and 80% training data. For transfer learning, the dataset is converted into image form to pass as input to the model. The parameter of the weight set is non-trainable and additional layers are added in transfer learning models. The results of the CNN-based transfer learning model are discussed below:

#### 4.3.1. VGG-19

**Figure 13:**
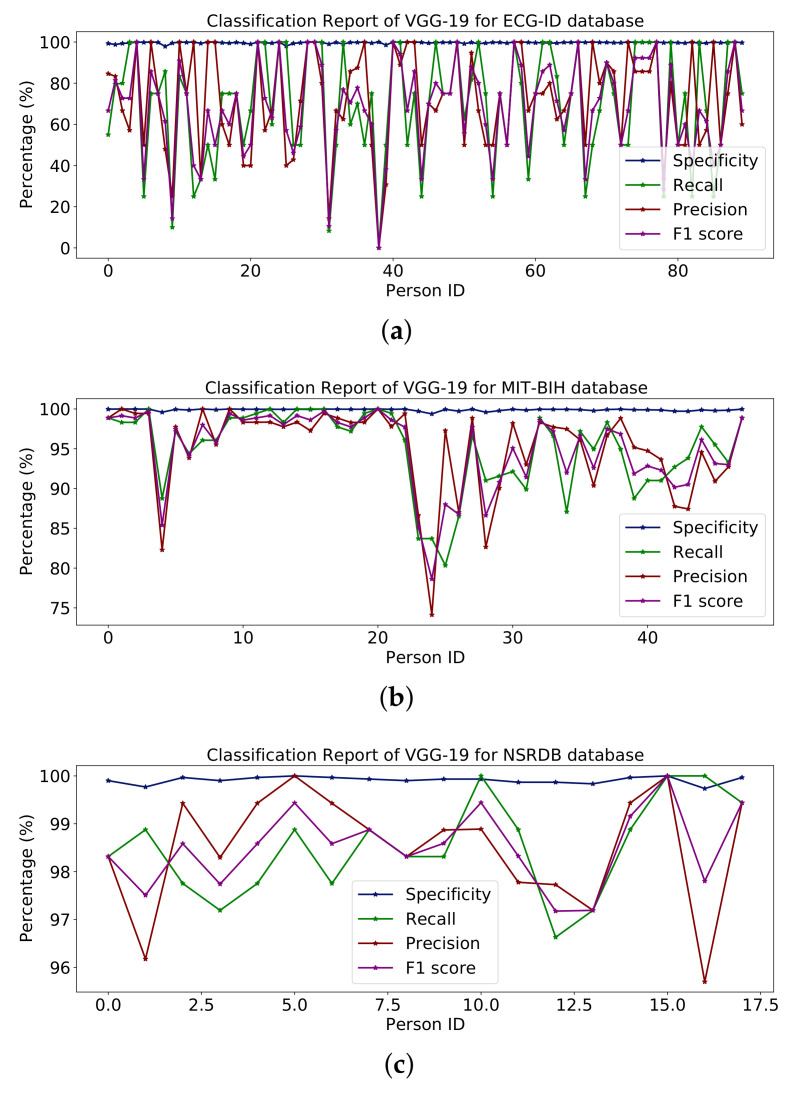
Classification report of VGG19 on the (**a**) ECG-ID, (**b**) MIT-BIH and (**c**) NSRDB databases.

Table 6 shows the whole model with some additional layers for classification. Figure 13 represents the percentages of specificity, recall, precision and F1-score for the databases ECG-ID, MIT-BIH and NSR-DB that include healthy and non-healthy signals, wherein the points in the graph line show the value of F1-score, precision, recall and sensitivity for each person. The mean value of specificity, recall, precision and F1-score using the ECG-ID database are 99.67%, 69.82%, 72.21% and 68.24%. For MIT-BIH, the mean score for specificity, recall, precision and F1-score is 99.89%, 94.99%, 95.14% and 95.01% and for NSR-DB, the mean values are 99.91%, 98.50%, 98.51% and 98.50%. In Figure 13, detailed reports of ECG-ID, MIT-BIH and NSR-DB are plotted. The accuracy achieved using VGG-19 on the mentioned databases is discussed in Table 9. The accuracies are 70.96%, 94.99% and 98.50%, respectively. Figure 13 illustrates the graph for the classification report of the VGG19 model on the ECG-ID, MIT-BIH and NSRDB databases.

#### 4.3.2. Inception Version 3

**Figure 14:**
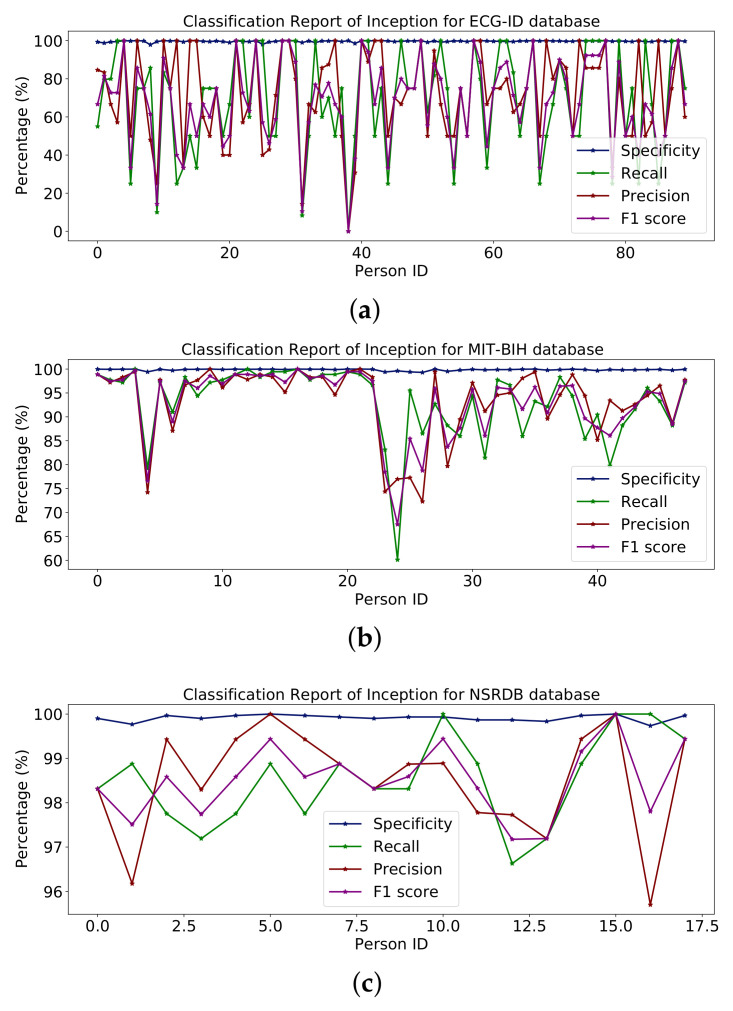
Classification report of Inception on (**a**) ECG-ID, (**b**) MIT-BIH and (**c**) NSRDB databases.

The whole model is shown in the summary in Table 7 along with some additional layers for classification. The report in Figure 14 shows the percentages of specificity, recall, precision and F1-score for the databases ECG-ID, MIT-BIH and NSR-DB, which include healthy and non-healthy signals; the points on the graph line represent the value of the F1-score, precision, recall and sensitivity for each person. Using the ECG-ID database, the mean values of specificity, recall, precision and F1-score are 99.67%, 69.82%, 72.21% and 68.24%, respectively. The mean values for specificity, recall, precision and F1-score for MIT-BIH are 99.85%, 93.16%, 93.40% and 93.17%, respectively, while for NSR-DB, they are 99.91%, 98.50%, 98.51% and 98.50%. The detailed reports of ECG-ID, MIT-BIH and NSR-DB were all plotted in Figure 14. The accuracy obtained with Inception version 3 on the mentioned database is reported in the results in Table 9. Accuracy rates are 70.96%, 93.16% and 98.50%, respectively. The graph for the classification report of the Inception version 3 model is shown in Figure 14.

#### 4.3.3. ResNet-152

**Figure 15:**
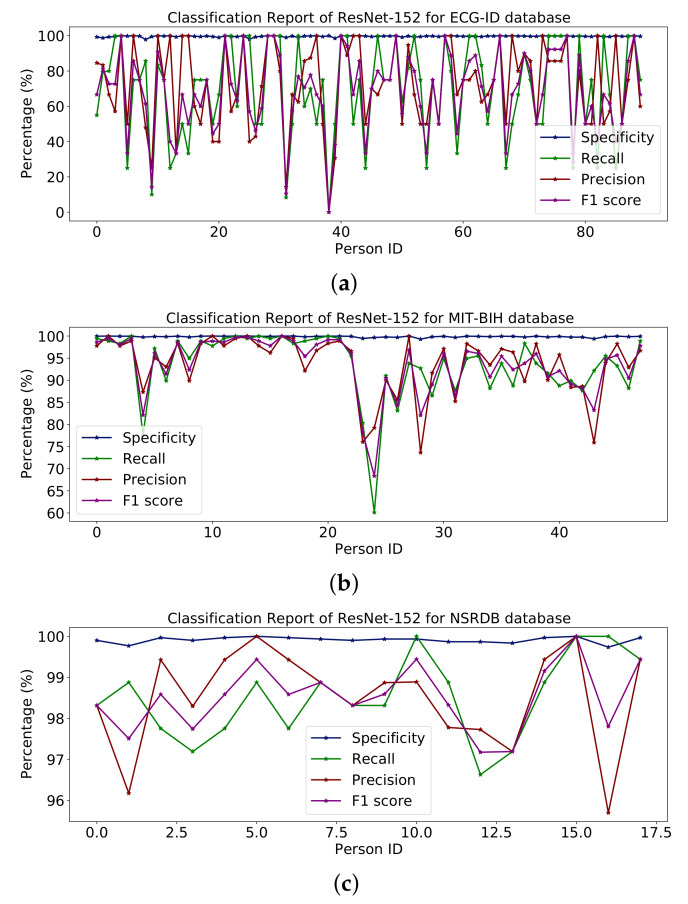
Classification report of ResNet-152 on (**a**) ECG-ID, (**b**) MIT-BIH and (**c**) NSRDB databases.

Table 8 shows the entire model with some additional classes for classification. Figure 15 shows percentages for specificity, recall, accuracy and F1 scores for the ECG-ID, MIT-BIH and NSR-DB databases including healthy and non-healthy ECG signals, wherein the plot lines show the value of the F1 score, accuracy, memory and sensitivity for each person. The means of specificity, recall, accuracy and F1 score obtained using the ECG-ID database were 99.67%, 69.82%, 72.21% and 68.24%. For MIT-BIH, the mean scores for specificity, recall, accuracy and F1 scores were 99.86%, 93.56%, 93.71% and 93.53%, and for NSR -DB, the mean values were 99.91 %, 98.50 %, 98.51 % and 98.50 %. In Figure 15, detailed reports of ECG-ID, MIT-BIH and NSR-DB are plotted. The accuracy obtained using ResNet-152 on the mentioned database is reported in Table 9. The accuracy is 70.96%, 93.56% and 98.50%, respectively.

The results reported for different databases using the ResNet-152 model are shown in Figure 15.

#### 4.3.4. Results Comparison VGG-19, ResNet-152 and Inception Version 3

All the transfer learning models give approximately the same result on the mentioned database. This section presents a comparison of the transfer learning model using different models and databases. The mean values of specificity, recall, precision and F1-score on models VGG-19, ResNet-152 and Inception version 3 using ECG-ID are 99.67%, 68.82%, 72.21% and 68.24%, for MIT-BIH, are 99.86%, 93.90%, 93.40% and 93.90% and for NSR-DB are 99.91%, 98.50%, 98.51% and 98.50%. The average accuracy value of VGG-19, ResNet-152 and Inception version 3 on the ECG-ID, MIT-BIH and NSR-DB databases is 88.15%, 87.67% and 87.54%. By analysis, it was determined that the average value of VGG-19 is better than those of ResNet-152 and Inception version 3. The average accuracy of VGG-19 is 0.48% greater than that of ResNet-152 and 0.61% better than that of Inception version 3. Furthermore, the ResNet-152 is 0.13% better than Inception version 3.

#### 4.3.5. Results for Proposed Models

The proposed model incorporates DWT and 1D-CRNN features. The best features selected from DWT based on their results, Coif5 and Haar wavelet give the best results on the random forest classifier (RFC). The model was trained using LSTM with 1D-CNN, the features extracted from the dense layer before the output layer, 128 features extracted from the dense layer and 256 features extracted from the DWT wavelet are combined to form hybrid features. The hybrid features are classified using the Random Forest classifier and the proposed method is shown in Table 10.

**Figure 16:**
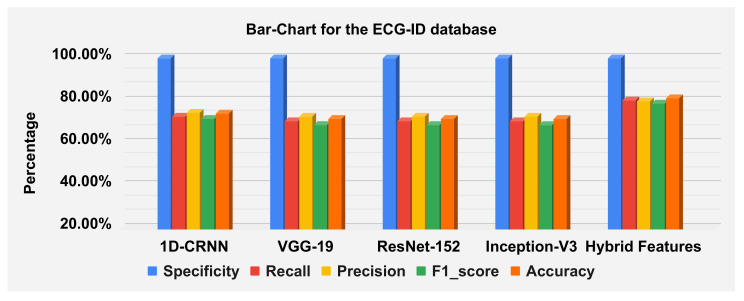
Barplot for the ECGID database using a confusion matrix. Blue, red, yellow, green and orange bars represent the mean specificity, recall, precision, F1 score and accuracy, respectively.

**Figure 17:**
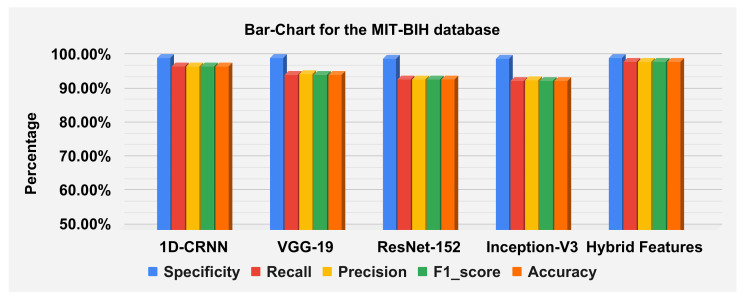
Barplot for MIT-BIH database using a confusion matrix. Blue, red, yellow, green and orange bars represent the mean specificity, recall, precision, F1 score and accuracy, respectively.

**Figure 18:**
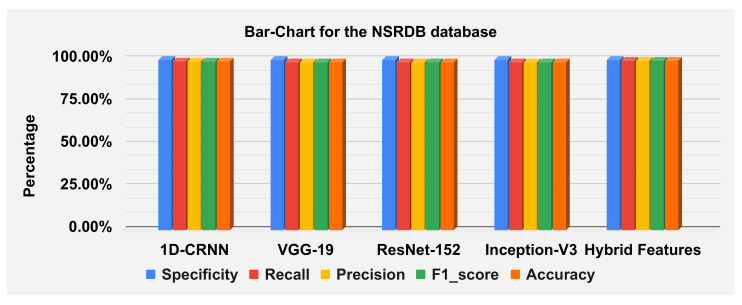
Barplot for NSRDB database using a confusion matrix. Blue, red, yellow, green and orange bars represent the mean specificity, recall, precision, F1 score and accuracy, respectively.

The hybrid features’ complexity analysis was performed on four different databases: ECG-ID, MIT-BIH, NSRDB and a combined dataset. The total parameters for ECG-ID, MIT-BIH, NSRDB and the combined dataset were 587,650, 573,232, 569,362 and 587,164, respectively. The training times for ECG-ID, MIT-BIH, NSRDB and the combined dataset were 1 min, 9 min, 4 min and 13 min and 12 s, respectively. The testing times for ECG-ID, MIT-BIH, NSRDB and the combined dataset are 0.77 ms, 0.53 ms, 1.01 ms and 0.60 ms, respectively. For a detailed analysis of the results, the barplot is shown in the figures for all datasets. The results contain the mean value of the F1 score, precision, recall and specificity and accuracy. These are plotted using bar plots. In Figure 16, the blue bar represents the mean specificity, the red bar represents the mean recall, the gray bar represents the mean precision, the yellow bar represents the mean f1-score and the green bar represents the accuracy. The models are represented on the x axis, and the percentage values are represented on the y axis. Figure 16 represents the ECG-ID database results, Figure 17 represents the MIT-BIH database results and Figure 18 represents the NSRDB database results.

**Figure 19:**
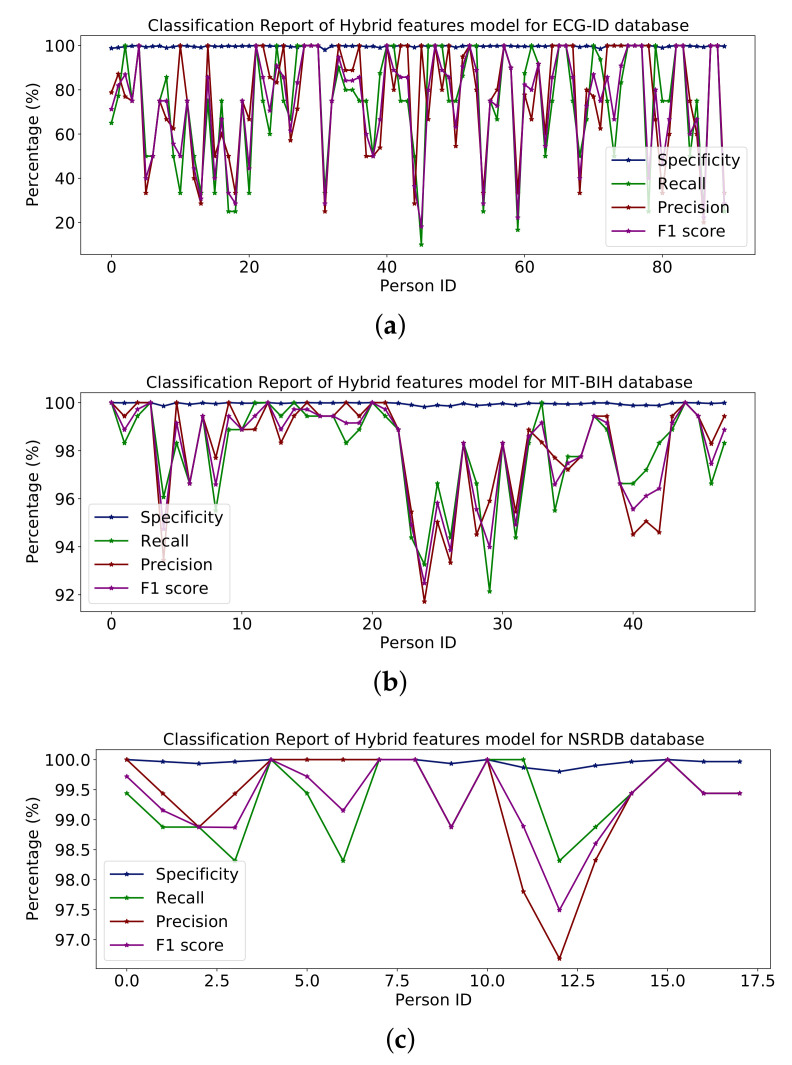
Classification report of hybrid on the (**a**) ECG-ID, (**b**) MIT-BIH and (**c**) NSRDB databases.

Figure 19 illustrate the classification report of each subject. The classification report contains the values for specificity, recall, precision and F1-score these can analyze on MIT-BIH, ECG-ID and NSR-DB databases. The mean values of specificity, recall, precision and F1-score using the proposed hybrid features’ base model on the ECG-ID are 99.78%, 79.70%, 79.50% and 78.12% for MIT-BIH are 99.97%, 98.81%, 98.83% and 98.82% and for NSR-DB databases are 99.97%, 99.62%, 99.62% and 99.62%, respectively. Figure 16, Figure 17 and Figure 18 represent the bar plots for the three databases. The comparison of different models such as CNN, VGG19, ResNet-152, Inception version 3 and the proposed hybrid features base model was analyzed on the MIT-BIH, ECG-ID and NSR-DB datasets. Thus, the proposed model gives a comparatively better result instead of the other transfer learning and CNN model base approach.

The ECG-ID dataset is collected from 90 subjects, which contains 310 recordings for the 90 subjects. Some of the subjects have ten samples, some have five samples, and some have two samples, so these are imbalanced data which can affect the performance of the training model. The performance decrease is because of those subjects having a small number of samples compared to the subjects with a large number of samples. Because of this non-scalability issue of the ECG-ID dataset, it is unlikely that this could be addressed using the current ECG-based techniques. The results are shown in Table 10 and the line graph for the ECG-ID database is shown using models VGG-19, ResNet-152, Inception version 3 and hybrid features-based model is shown in Figure 13, Figure 14, Figure 15 and Figure 19, respectively. When evaluating the performance of multi-class classification models, evaluation metrics such as accuracy, precision, recall, F1-score and specificity, are commonly used, as shown in Figure 10.

The accuracies of the proposed model on the above three datasets are 80.64%, 98.81% and 99.62%, and 98.24% on the combined dataset. The results for DWT-based features, 1D-CRNN-based features and proposed models’ hybrid-based features classification was discussed in Table 10. The average score of specificity, recall, precision and f1-score on the ECG-ID, MIT-BIH and NSR-DB database using a hybrid features-based model are 99.89%, 90.38%, 91.33% and 90.19%, respectively. By applying the transfer learning approach, VGG-19 gives better results compared to the Inception version 3 and ResNet-152. The average accuracy score of VGG-19 for all databases is 88.15% and for the hybrid feature-based model is 93.02%. Thus, the proposed model is 4.87% better than the transfer learning approach.

### 4.4. Ablation Analysis

We performed feature analysis on multiple QRS, the result for which is shown in Table 11. Empirically, we found that the accuracy of the combined dataset increased when we increased the number of QRS due to the repetitive tendency of RNN; it is probable that the RNN model’s repeating propensity helps it better understand patterns and correlations within the data, resulting in higher accuracy. However, in the case of the ECG-ID database, the consecutive QRS complex does not perform well, the small number of ECG samples in the dataset is an issue and an increasing number of QRS complexes does not result in a substantial gain in accuracy. The dataset with unbalanced samples and a higher number of classes does not reflect the improvement in biometric recognition performance. This study does not involve the analysis of arrhythmia, however, the variability among various rhythms reflected in arrhythmic patients can affect the performance of the biometric recognition system. To incorporate this, we proposed that the single QRS complex is enough for biometric recognition with less space complexity, as presented in Table 12. Similarly the time complexity analysis is presented in Table 13. Furthermore, we performed further experimentation with more than a single QRS complex, in order to analyze the empirical performance of our model. The increase in the number of consecutive QRS segments improves the recognition performance for the biometric recognition of arrhythmic patients as well as for healthy subjects. However, for the ECG-ID dataset of healthy subjects with a lesser number of segments and more classes, increasing the number of QRS complexes further reduces the emotion recognition performance. These results are summarized in Table 11.

### 4.5. Comparative Analysis

Table 14 discusses the experimental results of the proposed model with the previous related work.

In [22], that work, signals are decomposed to the multilevel for analysis and then 1D-CNN is applied to the ECG-ID dataset with 10 subjects containing two records and NSRDB [4] with 18 subjects. In [56], the MIT-BIH arrhythmia database as well as the delayed long short-term memory (DLSTM) were used to predict heartbeats from five classes. The model delays until the cross-ponding measure values are acquired. This model is only for five classes, but our proposed model identifies 156 subjects in a combined dataset. In [42], a method was proposed for converting ECG signals into scalogram images using the continuous wavelet transform (CWT) and then extracting two-dimensional features using the discrete wavelet transform (DWT) and passing them to the CNN model. Ref. [25], in their work, generated an electrocardiomatrix (EKM), which is a matrix that is composed of several aligned R peaks from an EKG trace and then transformed. Furthermore, afterwards, CNN is applied for further evaluation. The method in [33] was introduced, namely bi-directional gated recurrent units, which is a variant of LSTM. BGRU gives good performance on time series data. The nine consecutive heartbeats are passed through the model as input. However, as compared to our proposed method’s single sample with PQRS and T points, it is passed as input to the network. Thus, the method in [33] is not efficient and takes a significant amount of time for identification compared to our proposed method. In Ref. [34], a method was introduced that extracts parallel features from LSTM and 1D-CNN, and then both features extracted are merged and transferred to the classifier as a heatmap representation of an image. The article [32] used pre-configured deep convolutional neural networks for Biometrics from ECG Signals, which resulted from the different datasets discussed in Table 14. The non-fiducial graph regularized non-negative matrix factorization (GNMF)-based features method was introduced in [53]; however, this method will classify irrelevant data and may take longer to identify. When compared to our proposed model, our method is more robust and can identify a person with a single beat. In the proposed method [19], with only five heartbeats, it is possible to attain high subject recognition on ECG signals. The principal component analysis network (PCANet) method employs a straightforward deep learning technique to extract high-level features from the input signal. However, our proposed model was applied to all subjects in the ECG-ID database with all recordings for person identification. Compared to the previous studies, our proposed model was applied to a large dataset segmented sample of the ECG signal for identification. The samples of all three datasets were combined to make large datasets, whilst the combined dataset approach was not attempted in previous studies.

## 5. Conclusions

In this study, electrocardiogram signals were used as a biometric option to fill the security gaps left by both traditional identifying methods and other recently developed biometric technologies. The proposed method demonstrates the utility of wavelet and 1D-CNN-based features in comparison to fiducial features. The proposed methodology was tested on both publicly available datasets with healthy subjects as well as the dataset of ECG with arrhythmia for biometric recognition with promising results. The ECG is particularly useful in biometric identification applications because the heart is located inside the body and moves while a subject is alive. The signal frequency domain analysis has a large impact on noise. Because the ECG is sensitive to noise, various studies have used noise-resistant time–frequency transformations, with the proposed approach combining DWT, LSTM and 1D-CNN providing superior performance for ECG biometric classification. Various transfer learning models, including VGG-19, ResNet-152 and Inception version 3, were used to compare the performance of the proposed approach. The system is best served by the optimal choice of the Coif5 wavelet coefficient with level 4 decomposition, which was analyzed and compared, as was the performance of the different wavelet family functions used for extracting features, and the 1D-CNN + LSTM extracted features were concatenated with the Coif5 wavelet-based feature for evaluation. The combined features technique (DWT+ LSTM + 1D-CNN) gives a better result compared to the transfer learning approach. The evaluation of the combined features technique was performed on a large dataset. The proposed model can also be evaluated for other physiological signal-based biometric modalities and compared with other 1D-CNN models in future work.

## Figures and Tables

**Table 1:** Details of the ECG databases, including signal length, sampling frequency, resolution, published date and total records.

Database Name	Published	No. of Subjects	Signal (Time-Period)	Sampling Frequency	Total Records	Resolution
ECG-ID database	6 March 2014	90	20 s	500 Hz	310	12 bit
MIT-BIH database	24 February 2005	48	30 min	360 Hz	48	11 bit
NSRDB database	3 August 1999	18	1 h 30 min	128 Hz	18	11 bit

**Table 2:** Summary of the 1D-CRNN architecture with the units with learnable parameters.

Serial No.	Layer	Output Shape	Parameters
1	LSTM	(0, 94, 128)	66,560
2	LSTM	(0, 94, 64)	49,408
3	Conv1D	(0, 94, 32)	6176
4	Conv1D	(0, 94, 64)	10,304
5	Conv1D	(0, 94, 32)	16,416
6	Dropout	(0, 94, 32)	0
7	Maxpool	(0, 47, 32)	0
8	Dropout	(0, 47, 32)	0
9	Flatten	(0, 1504)	0
11	Dense	(0, 256)	385,280
**12**	**Dense**	**(0, 128)**	**32,896**
13	Dense	(0, No. of classes)	6192

**Table 3:** Results of conventional DWT features using the SVM classifier with the RBF kernel on databases such as ECG-ID, MITBIH and NSRDB.

Database Name	Haar	db2	bio6.8	sym5	Coif5
ECGID	53%	54%	54%	54.51%	59%
MIT-BIH	88.93%	89.34%	89.71%	89.36%	89.96%
NSRDB	97.81%	97.97%	97.90%	97.97%	98%

**Table 4:** Results of conventional DWT features using KNN classifier on databases such as ECG-ID, MITBIH and NSRDB.

Database Name	Haar	db2	bio6.8	sym5	Coif5
ECGID	65.32%	63.70%	55.80%	61.61%	64.19%
MIT-BIH	96.70%	96.56%	96.20%	96.60%	96.65%
NSRDB	99.0%	98.97%	98.65%	98.78%	98.75%

**Table 5:** Results of conventional DWT features using random forest classifier on databases such as ECG-ID, MITBIH and NSRDB.

Database Name	Haar	db2	bio6.8	sym5	Coif5
ECGID	74.19%	73.06%	64.83%	70.80%	73.04%
MIT-BIH	97.70%	97.69%	96.67%	97.12%	97.60%
NSRDB	99.15%	98.93%	98.90%	98.03%	99.15%

**Table 6:** Detailed architecture of the fine-tuned VGG19 model with learnable parameters.

Serial No.	Layer (Type)	Output Shape	Learnable Parameters
1	VGG-19	(0, 3, 3, 512)	20,024,384
2	Dense	(0, 512)	262,656
3	Dense	(0, 256)	131,328
4	Dropout	(0, 256)	0
5	Dense	(0, 128)	32,896
6	Dense	(0, output)	6192

**Table 7:** The basic architecture of the Inception V3 model.

Serial No.	Layer (Type)	Output Shape	Learnable Parameters
1	Inception-V3	(0, 1, 1, 2048)	21,802,784
2	Dense	(0, 512)	262,656
3	Dense	(0, 256)	131,328
4	Dropout	(0, 256)	0
5	Dense	(0, 128)	32,896
6	Dense	(0, output)	6192

**Table 8:** The architecture of the fine-tuned transfer learning model ResNet-152 model.

Serial No.	Layer (Type)	Output Shape	Learnable Parameters
1	ResNet-152	(0, 4, 4, 2048)	58,370,944
2	Dense	(0, 512)	262656
3	Dense	(0, 256)	131,328
4	Dropout	(0, 256)	0
5	Dense	(0, 128)	32,896
6	Dense	(0, Output)	6192

**Table 9:** Accuracy of pretrained models Vgg19, ResNet-152 and Inception version 3 on the ECG Scalogram images.

Database Name	VGG-19	ResNet-152	Inception Version 3
ECGID	70.96%	70.96%	70.96%
MITD	94.99%	93.56%	93.16%
NSRDB	98.50%	98.50%	98.50%

**Table 10:** Accuracy of the models 1D-CRNN, DWT+ random forest and DWT + 1D-CRNN + random forest on ECG segmented samples.

Database Name	1D-CRNN	DWT + Random Forest	DWT + 1D-CRNN + Random Forest	Training Samples	Testing Samples	Number of Classes
ECGID	73.55%	73.04%	80.64%	2480	620	90
MIT-BIH	97.50%	97.60%	98.81%	34,464	8544	48
NSRDB	99.06%	99.15%	99.62%	12,924	3204	18
Combined dataset	96.91%	96.28%	98.24%	49,868	12,368	156

**Table 11:** Comparison of various datasets with various consecutive QRS complexes against learnable parameters using the proposed methodology.

	1 QRS	2 QRS	3 QRS	4 QRS
ECG-ID dataset	80.64%	71.05%	65.28%	63.59%
MITBIH dataset	98.81%	99.83%	99.89%	99.85%
NSRDB dataset	99.62%	99.87%	99.91%	99.89%
Combined dataset	98.24%	98.4%	98.59%	99.03%

**Table 12:** Learnable parameters on various datasets with different consecutive QRSs.

	1 QRS	2 QRS	3 QRS	4 QRS
ECG-ID dataset	578,650	963,674	1,348,698	1,733,722
MITBIH dataset	573,232	958,256	1,343,280	1,728,304
NSRDB dataset	569,362	954,386	1,339,410	1,724,434
Combined Dataset	587,164	972,188	1,357,212	1,742,236

**Table 13:** Durations of the ECG testing times on various datasets with different consecutive QRSs.

	1 QRS	2 QRS	3 QRS	4 QRS
ECG-ID dataset	0.77 ms	0.10 ms	0.08 ms	0.05 ms
MITBIH dataset	0.53 ms	0.76 ms	0.74 ms	0.75 ms
NSRDB dataset	1.01 ms	1.40 ms	1.12 ms	0.07 ms
Combined Dataset	0.60 ms	0.81 ms	1.01 ms	0.83 ms

**Table 14:** Comparison of the proposed approach with the previous study.

Author	Dataset	Model Type	Performance
Bajare et al. [22]	NSRDB (number of subjects: 18)	1D-CNN + DWT	96.93%
	MIT-BIH		96.82%
	ECG-ID (number of subject: 10)		100 %
Benouis et al. [52]	ECG-ID	1D local difference pattern (1D-LDP)	91.11%
El et al. [42]	NSRDB	CWT + DWT + Deep learning	100%
	PTB		99.5%
	MIT-BIH arrhythmia		98.1%
	STDB		98.2%
Fuster et al. [25]	MITBIH	EKM + CNN	98.84%
	NSRDB [4]		99.53%
	PTB		89.8%
Li et al. [53]	ECG-ID	GNMF-based feature extraction	100%
	MITBIH	Non-fudicial method	100%
Singh et al. [54]	MIT-BIH	Heartbeat interval	87.37%
	IIT(BHU)	Features	92.88%
Byeon et al. [32]	PTB-ECG	PCANET	98.54%
	CU-ECG		90.42%
	MIT-BIH		84.78%
Wang et al. [55]	ECG-ID (two recordings)	DWT + S-AEs	95.1%
	ECG-ID (all recordings)		92.3%
	MIT-BIH		96.82%
Wang et al. [19]	ECG-ID (only five heartbeats)	PCANET-features	94.4%
Yildirim et al. [56]	MIT-BIH (5 no. of Classes)	DULSTM-WS2	99.25%
	MIT-BIH (5 no. of classes)	DBLSTM-WS3	99.39%
Zhang et al. [40]	NSRDB [4]	Multiscale 1D-CNN	95.1%
	MIT-BIH		91.1%
Zhang et al. [21]	Single-arm-ECG	Based on (DCNN)	98.4%
	Ear-ECG		91.1%
Lynn et al. [33]	ECG-ID (9-consecutive heartbeat input)	BGRU (Bi-directional gated)	98.60%
	MIT-BIH (9-consecutive heartbeat input)	Recurrent units	98. 40%
Proposed	ECD-ID	1D-CRNN+ DWT	80.64%
Methodology	MIT-BIH		98.81%
	NSRDB		99.62%
	Combined dataset		98.24%

## Data Availability

Not applicable.
